# The Therapeutic Effect of PLAG against Oral Mucositis in Hamster and Mouse Model

**DOI:** 10.3389/fonc.2016.00209

**Published:** 2016-10-17

**Authors:** Ha-Reum Lee, Nina Yoo, Joo Heon Kim, Ki-Young Sohn, Heung-Jae Kim, Myung-Hwan Kim, Mi Young Han, Sun Young Yoon, Jae Wha Kim

**Affiliations:** ^1^Cell Factory Research Center, Division of Systems Biology and Bioengineering, Korea Research Institute of Bioscience and Biotechnology, Daejeon, South Korea; ^2^ENZYCHEM Lifesciences, Daejeon, South Korea; ^3^Department of Pathology, Eulji University School of Medicine, Daejeon, South Korea; ^4^Division of Gastroenterology, Department of Internal Medicine, University of Ulsan College of Medicine, Asan Medical Center, Seoul, South Korea; ^5^Ju-Sikyung Liveral Arts College, Pai Chai University, Daejeon, South Korea; ^6^Department of Functional Genomics, University of Science and Technology, Daejeon, South Korea

**Keywords:** PLAG, neutrophil, oral mucositis, chemotherapy, cachexia

## Abstract

Chemotherapy-induced mucositis can limit the effectiveness of cancer therapy and increase the risk of infections. However, no specific therapy for protection against mucositis is currently available. In this study, we investigated the therapeutic effect of PLAG (1-palmitoyl-2-linoleoyl-3-acetyl-rac-glycerol, acetylated diglyceride) in 5-fluorouracil (5-FU)-induced oral mucositis animal models. Hamsters were administered 5-FU (80 mg/kg) intraperitoneally on days 0, 6, and 9. The animals’ cheek pouches were then scratched equally with the tip of an 18-gage needle on days 1, 2, and 7. PLAG was administered daily at 250 mg/kg/day. PLAG administration significantly reduced 5-FU/scratching-induced mucositis. Dramatic reversal of weight loss in PLAG-treated hamsters with mucositis was observed. Histochemical staining data also revealed newly differentiated epidermis and blood vessels in the cheek pouches of PLAG-treated hamsters, indicative of recovery. Whole blood analyses indicated that PLAG prevents 5-FU-induced excessive neutrophil transmigration to the infection site and eventually stabilizes the number of circulating neutrophils. In a mouse mucositis model, mice with 5-FU-induced disease treated with PLAG exhibited resistance to body-weight loss compared with mice that received 5-FU or 5-FU/scratching alone. PLAG also dramatically reversed mucositis-associated weight loss and inhibited mucositis-induced inflammatory responses in the tongue and serum. These data suggest that PLAG enhances recovery from 5-FU-induced oral mucositis and may therefore be a useful therapeutic agent for treating side effects of chemotherapy, such as mucositis and cachexia.

## Introduction

Oral mucositis is a common complication of chemotherapy and is often accompanied by erythema, ulceration, pain, weight loss, and delayed remission (1). Chemotherapy-induced mucositis occurs in 40–70% of patients and can limit the anticancer effects of chemotherapy, thus extending the therapeutic period and potentially reducing survival (2). Mucositis is defined as damage and inflammation in the mucous membranes lining the gastrointestinal tract (3). Chemotherapy can induce epithelial damage, which can then lead to a significant increase in inflammatory cytokine and chemokine secretion and activation of neutrophil extravasation (4). Neutrophils are the first leukocytes recruited to infected tissue, where they function to eliminate pathogens (5). Neutrophils destroy pathogen cells *via* phagocytosis, degranulation, and the neutrophil extracellular trap (NET) (6, 7). Although neutrophils play a critical role in the innate immune system, excessive transmigration of neutrophils can result in neutropenia and severe inflammation in the host tissue (8). Mucositis may, therefore, limit the effectiveness of chemotherapy, resulting in an extension of the treatment period and perhaps a decrease in patient survival (9).

The anti-metabolite drug 5-fluorouracil (5-FU) is widely used for the treatment of solid tumors, including colorectal and breast cancers (10). 5-FU is an analog of uracil that non-specifically blocks DNA synthesis, thus inhibiting cell division (11). PLAG (1-palmitoyl-2-linoleoyl-3-acetyl-rac-glycerol) is an acetylated form of diacylglycerol and a mono-acetyl-diglyceride that was first isolated from the antlers of sika deer (12). PLAG can be chemically synthesized using glycerol, palmitic acid, and linoleic acid, and the synthetic form has been confirmed to be identical with the naturally isolated form (13). In a previous study, PLAG was shown to exert a therapeutic effect with pegfilgrastim to treat chemotherapy-induced neutropenia by modulating neutrophil transmigration (14). Because PLAG regulates neutrophil transmigration, we hypothesized that PLAG administration would ameliorate the sequelae associated with oral mucositis.

To characterize the effect of PLAG on oral mucositis, we established oral mucositis models in mice and hamsters in which the disease is induced by 5-FU administration and scratching of the cheek pouches and/or tongue with the tip of an 18-gage needle (15). The scratching increases the risk of infection and thus induces neutrophil recruitment into the inflamed tissues. Using these models, we found that PLAG administration significantly reduced the symptoms of oral mucositis. PLAG reversed the weight loss, ulceration, and severe inflammation associated with 5-FU/scratching-induced mucositis. These data indicate that PLAG could be therapeutically useful in reducing the complications associated with chemotherapy, such as oral mucositis and cachexia, and thus may be an excellent supplementary agent for anticancer therapy.

## Materials and Methods

### Animal Experiments

Male Syrian Golden Hamsters were obtained from Japan SLC (Shizuoka Prefecture, Japan). The hamsters were 6 weeks old, weighed 120–140 g, and were maintained under specific pathogen-free conditions. BALB/c mice were obtained from Koatech Co. (Pyongtaek, Republic of Korea) and maintained under specific pathogen-free conditions. The mice were 6–8 weeks of age and weighed 20–22 g at the time of the experiments.

All animal experimental procedures were performed in accordance with the Guide for the Care and Use of Laboratory Animals (Institute of Laboratory Animal Resources).

### Ethics Statement

All experiments were approved by the Institutional Review Committee for Animal Care and Use of KRIBB (Korea Research Institute of Bioscience and Biotechnology, Daejeon, Republic of Korea).

### Oral Mucositis Experimental Models

Hamsters were intraperitoneally administered 5-FU (Sigma Aldrich, MO, USA) at 80 mg/kg on days 0, 6, and 9. For the scratching group, hamsters were anesthetized with 2,2,2-tribromoethanol (150 mg/kg, Sigma Aldrich) by intraperitoneal injection, and then a 1-cm^2^ area of each cheek pouch was scratched with the tip of an 18-gage needle (Koreavaccine, Aansan, Republic of Korea) at an equal force and depth on days 1, 2, and 7. The scratching was blinded to the treatment groups (*n* = 3 per group). PLAG (Enzychem Lifesciences Co., Daejeon, Republic of Korea) was then administered orally at 250 mg/kg/day.

Mice were intraperitoneally administered 5-FU (Sigma Aldrich) at 100 mg/kg on day 0. For the scratching group, mice were anesthetized with 2,2,2-tribromoethanol (150 mg/kg, Sigma Aldrich) by intraperitoneal injection, and then a 0.5-cm^2^ area of the tongue was scratched using the tip of an 18-gage needle (Koreavaccine) at an equal force and depth on days 7 and 8. The scratching was blinded to the treatment groups (*n* = 5 per group). PLAG (Enzychem Lifesciences) was then administered orally at 250 mg/kg/day beginning on day 7.

### Assessment of Mucositis

Mucositis was scored by a blinded investigator using published criteria based on the following parameters: erythema, vasodilation, erosion, bleeding, fibrosis, and ulcers (Table 1) (16).

**Table 1:** **Mucositis scoring criteria [from Sonis et al. (16)]**.

Score	Parameter
0	Completely healthy cheek pouch with no erosion or vasodilatation
1	Presence of erythema, but no evidence of erosion in the cheek pouch
2	Severe erythema, vasodilation, and surface erosion
3	Formation of ulcers in one or more faces of the mucosa, but not affecting more than 25% of the surface area of the cheek pouch, as well as severe erythema and vasodilatation
4	Cumulative formation of ulcers of about 50% of the surface area of the cheek pouch
5	Complete ulceration of the cheek pouch mucosa, in which the fibrosis makes oral mucosa exposure difficult

### Hematoxylin and Eosin Staining

Samples of cheek pouch tissue were obtained on day 13, fixed in 10% buffered formalin for 24 h, embedded in paraffin, and sectioned at a thickness of 4 μm. The tissue sections were stained with hematoxylin and eosin (H&E), and the degree of inflammatory cell infiltration was assessed. Sections were observed under a light microscope (Olympus, Tokyo, Japan).

### Peripheral Blood Analysis

For hamsters, whole blood was collected from the orbital sinuses using capillary tubes (Kimble Chase Life Science and Research Products LLC, FL, USA) and collection tubes (Greiner Bio-One International, Frickenhausen, Germany) containing K3E-K3EDTA. Blood cells were enumerated by CBC analysis using a BC 5300 Mindray analyzer (Shenzhen Mindray Bio-medical Electronics, China).

### ELISA

The concentrations of interleukin (IL)-6, tumor necrosis factor (TNF), and IL-1β in the supernatant of homogenized tongue, and serum samples were measured using a mouse IL-6 ELISA kit, mouse TNF ELISA kit, and mouse IL-1β ELISA kit (all from BD Bioscience, NJ, USA), respectively, according to the manufacturer’s instructions. The cytokine levels were estimated by interpolation from a standard curve generated using an ELISA reader (Molecular Devices) at a wavelength of 450 nm.

### Statistical Analyses

Statistical analyses were performed using paired Student’s *t*-tests. Differences were considered statistically significant at *p* < 0.001, *p* < 0.01, and *p* < 0.05.

## Results

### PLAG Alleviated the Symptoms of Severe Mucositis in the Hamster Model

**Figure 1:**
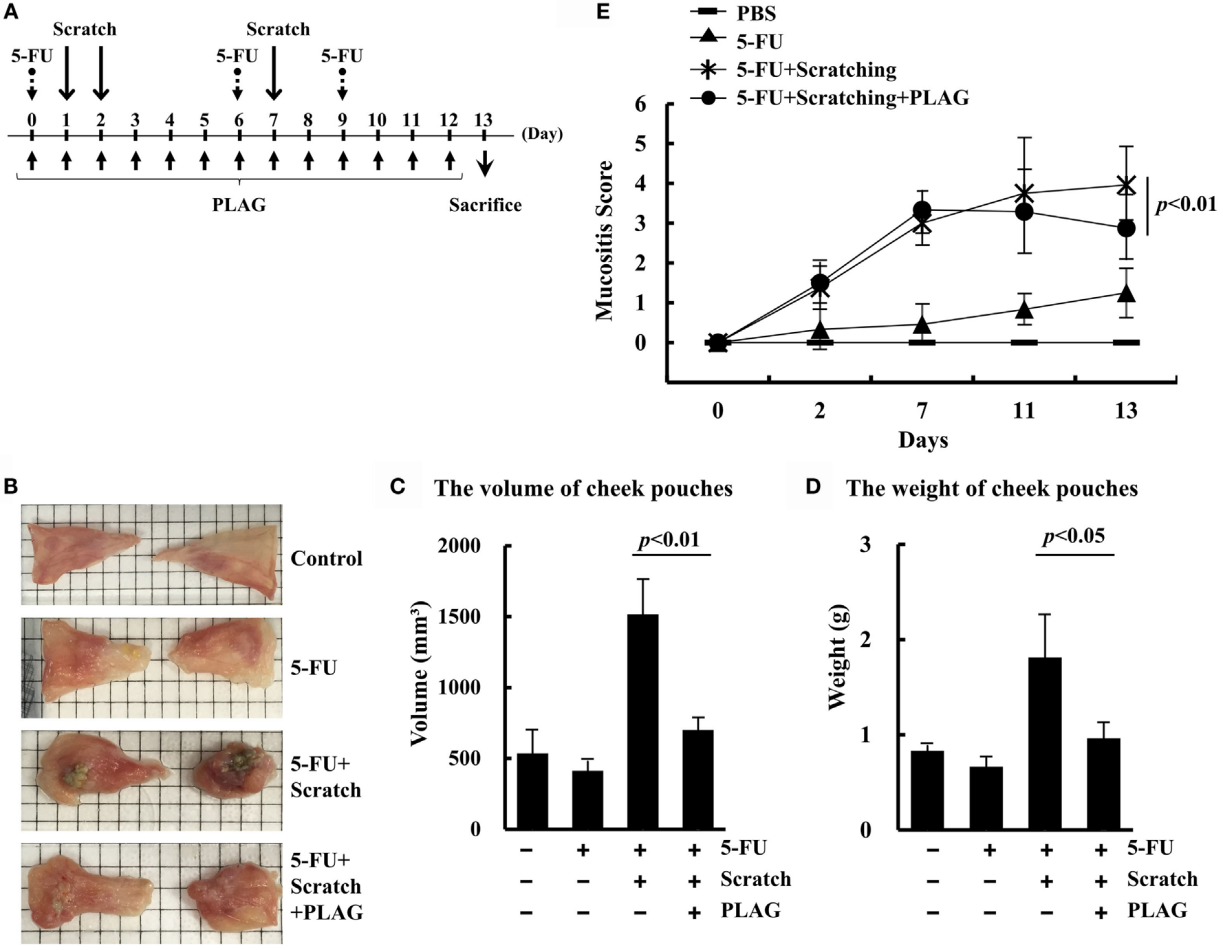
**The hamster mucositis model**. Hamsters were divided into four groups (*n* = 3 per group): (1) control group, (2) 5-FU treatment group, (3) scratching/5-FU treatment group, and (4) scratching/5-FU/PLAG treatment group **(A)**. 5-FU was administered intraperitoneally at 80 mg/kg on days 0, 6, and 9. PLAG was administered orally at 250 mg/kg/day. For the scratching model, hamsters were anesthetized and the cheek pouches were scratched with an equal force and depth on days 1, 2, and 7. The cheek pouches were isolated on day 13 **(B)**. The volume **(C)** and weight **(D)** of the isolated cheek pouches were measured. Mucositis scores were determined on days 2, 7, 11, and 13 **(E)**. 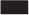, control; 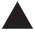, 80 mg/kg 5-FU; 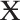, scratching with 80 mg/kg 5-FU; and 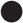, scratching with 80 mg/kg 5-FU and 250 mg/kg PLAG. Average values are shown, and the bars represent error ranges. Statistical significance was assessed using Student’s *t*-test.

To investigate whether PLAG has a therapeutic effect on 5-FU-induced mucositis in the hamster model, 5-FU was intraperitoneally administered on days 0, 6, and 9 (Figure 1A). The cheek pouches were scratched equally on days 1, 2, and 7, and PLAG was administered orally every day thereafter. To quantify the level of oral mucositis, the cheek pouches were isolated on day 13. The 5-FU/scratching group exhibited severe ulceration, fibrosis, and festering wounds (Figure 1B). PLAG treatment significantly decreased ulcer formation and diminished the degree of wound festering. No fibrosis was observed in the cheek pouches of PLAG-treated hamsters. Inflammation of cheek pouches induced by 5-FU/scratching was ameliorated by PLAG administration, which significantly decreased the volume and weight of isolated pouches (Figures 1C,D). PLAG administration also had an effect on the mucositis score (Figure 1E). Compared with the 5-FU/scratching group, PLAG-treated hamsters exhibited a lower mucositis score (*p* < 0.01). These data indicate that PLAG has a significant therapeutic effect against chemotherapy-induced oral mucositis.

### PLAG Administration Reversed Weight Loss in the Hamster Mucositis Model

**Figure 2:**
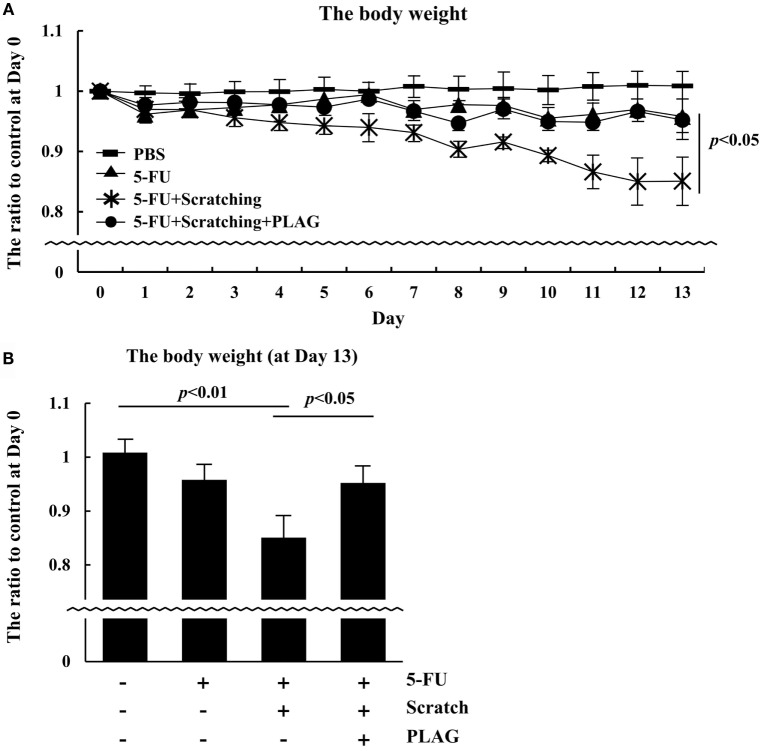
**Body weight in the hamster mucositis model**. **(A)** The groups of hamsters (*n* = 3 per group) were treated as described for Figure 1A, and body weight was recorded daily. **(B)** The body weight of hamsters was evaluated on day 13. 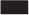, control; 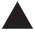, 80 mg/kg 5-FU; 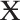, scratching with 80 mg/kg 5-FU; and 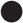, scratching with 80 mg/kg 5-FU and 250 mg/kg PLAG. Average values are shown, and the bars represent error ranges. Statistical significance was assessed using Student’s *t*-test.

Cancer patients usually experience a decline in body weight and difficulty in eating. This condition, known as cachexia, is associated with a poor therapeutic prognosis (17). To characterize the effect of PLAG on mucositis-associated cachexia, the body weight of hamsters was monitored. PLAG administration had a significant effect in preventing weight loss associated with 5-FU/scratching-induced mucositis (Figure 2A). By day 13, hamsters in the 5-FU/scratching group exhibited a 15% decline in body weight compared to controls (Figure 2B). Hamsters subjected to 5-FU/scratching and PLAG administration lost only 5% of their body weight. These results suggest that chemotherapy-associated cachexia may be prevented by PLAG administration.

### PLAG Administration Suppressed Mucositis-Induced Inflammation in Hamsters

**Figure 3:**
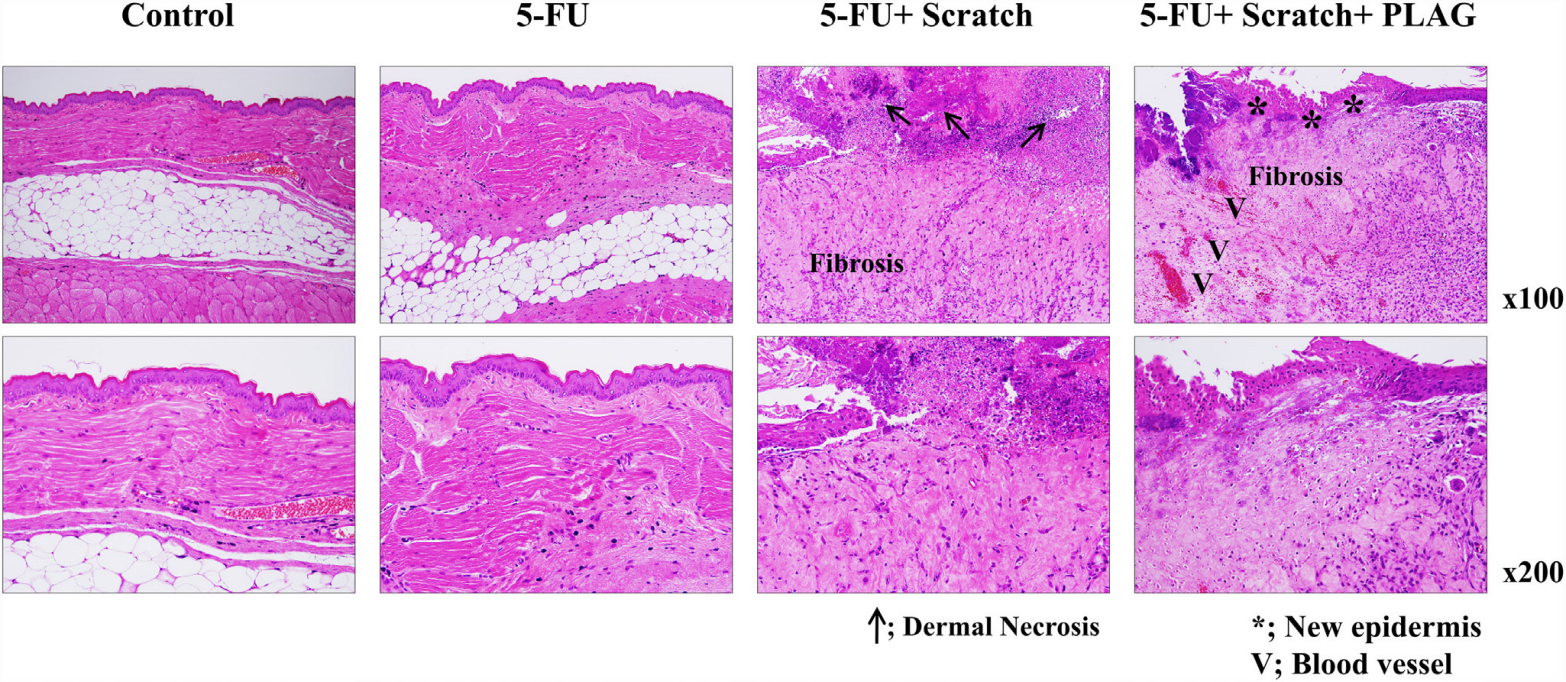
**Histopathologic analysis of hamster cheek pouches**. The groups of hamsters were treated as described for Figure 1A. After 13 days, the cheek pouches were isolated and stained with H&E. Tissue sections were examined under a light microscope (100× and 200×). Representative images of the sections are presented.

For histopathologic analysis, isolated hamster cheek pouches were stained with H&E. Hamsters in the 5-FU/scratching group exhibited dermal necrosis and severe fibrosis, with an increase in the thickness of the mucosal epidermis (Figure 3). Hamsters subjected to 5-FU/scratching and PLAG administration exhibited newly formed epidermis and blood vessels in the cheek pouches, indicative of recovery. These data also suggest that PLAG is useful for treating or preventing chemotherapy-induced oral mucositis.

### PLAG Administration Prevented 5-FU-Induced Excessive Neutrophil Transmigration into Scratched Tissues in the Hamster Mucositis Model

**Figure 4:**
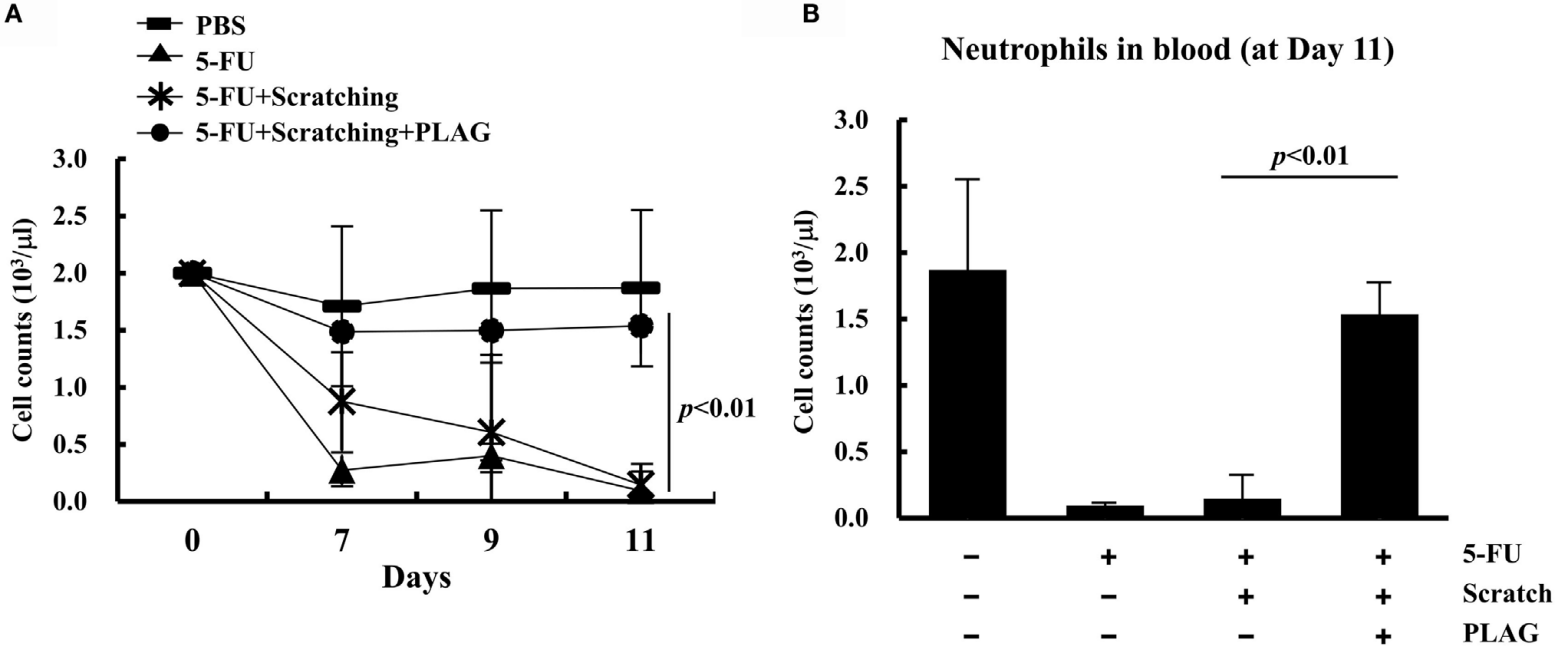
**Circulating neutrophils in the hamster mucositis model**. Groups of hamsters (*n* = 3 per group) were treated as described for Figure 1A. Whole blood was collected, and the number of circulating neutrophils was determined on days 0, 7, 9, and 11 **(A)**. Neutrophils were evaluated on day 11 **(B)**. 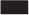, control; 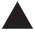, 80 mg/kg 5-FU; 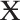, scratching with 80 mg/kg 5-FU; and 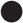, scratching with 80 mg/kg 5-FU and 250 mg/kg PLAG. Average values were calculated. The bars represent error ranges. Statistical significance was assessed using Student’s *t*-test.

Circulating blood cells were counted on days 7, 9, and 11. The neutrophil count was significantly reduced by 5-FU treatment and steadily declined in hamsters subjected to 5-FU/scratching (Figure 4A). Hamsters that received PLAG with 5-FU/scratching had a greater number of neutrophils than hamsters treated with 5-FU alone or 5-FU/scratching alone. Moreover, the number of circulating neutrophils in 5-FU/scratching hamsters treated with PLAG was similar to that of control animals (Figure 4B). These findings indicate that PLAG plays a role in reversing the decline in the number of circulating neutrophils associated with chemotherapy-induced neutropenia.

### PLAG Administration Reversed Weight Loss and Cured Ulceration in the Mouse Mucositis Model

**Figure 5:**
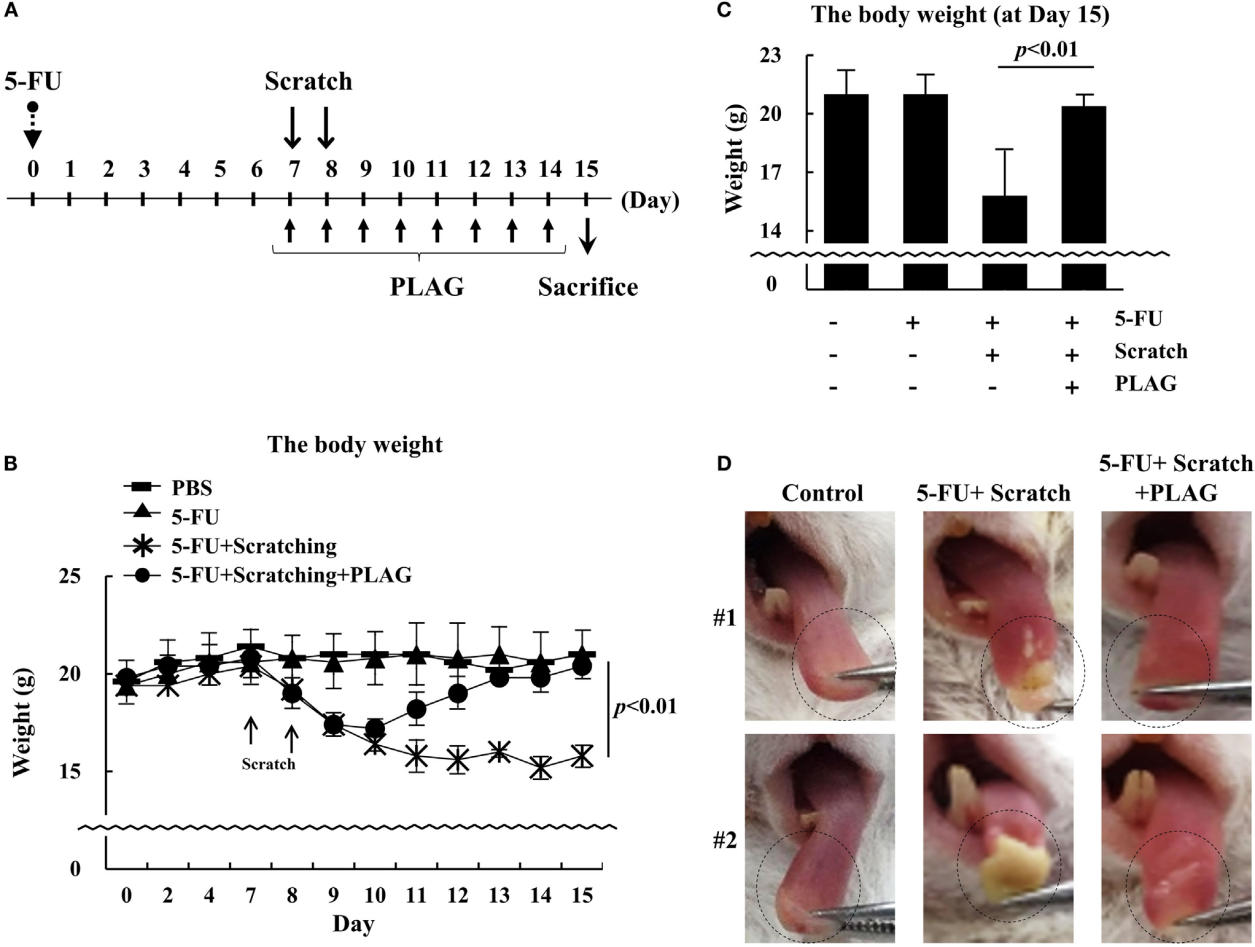
**Changes in body weight of mice in the 5-FU-induced mucositis model**. Mice were divided into four groups (*n* = 5 per group): (1) control, (2) 5-FU treatment, (3) scratching/5-FU treatment, and (4) scratching/5-FU/PLAG treatment **(A)**. 5-FU was administered intraperitoneally at 100 mg/kg on day 0. For the scratching group, mice were anesthetized and the tongue was scratched equally on days 7 and 8. PLAG was orally administered at 250 mg/kg/day beginning on the day of scratching for 8 days. Body weight was recorded daily **(B)**. 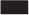, control; 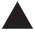, 100 mg/kg 5-FU; 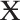, scratching with 100 mg/kg 5-FU; and 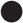, scratching with 100 mg/kg 5-FU and 250 mg/kg PLAG. The body weight of mice was evaluated on day 15 **(C)**. The tongues were evaluated on day 15 **(D)**. Average values were calculated, and the bars represent error ranges. Statistical significance was assessed using Student’s *t*-test.

Treatment of hamster subjected to 5-FU/scratching with PLAG prevented the progression of oral mucositis. To investigate the therapeutic effect of PLAG in mice, the animals were administered 5-FU on day 0, and after 7 days, PLAG was orally administered with scratching of the tongue (Figure 5A). After scratching, the mice exhibited a rapid and significant loss of body weight (Figure 5B). However, in mice also treated with PLAG, the body weight returned to the control level by day 15 (Figure 5C).

Figure 5D shows that 5FU/scratching resulted in severe ulceration of the tongue. In PLAG-treated mice, however, the scratching did not lead to ulceration. These data suggest that PLAG is a useful therapeutic agent for treating side effects of chemotherapy such as mucositis and cachexia.

### PLAG Administration Diminished Mucositis-Induced Inflammatory Response in the Tongue and Serum of Mice

**Figure 6:**
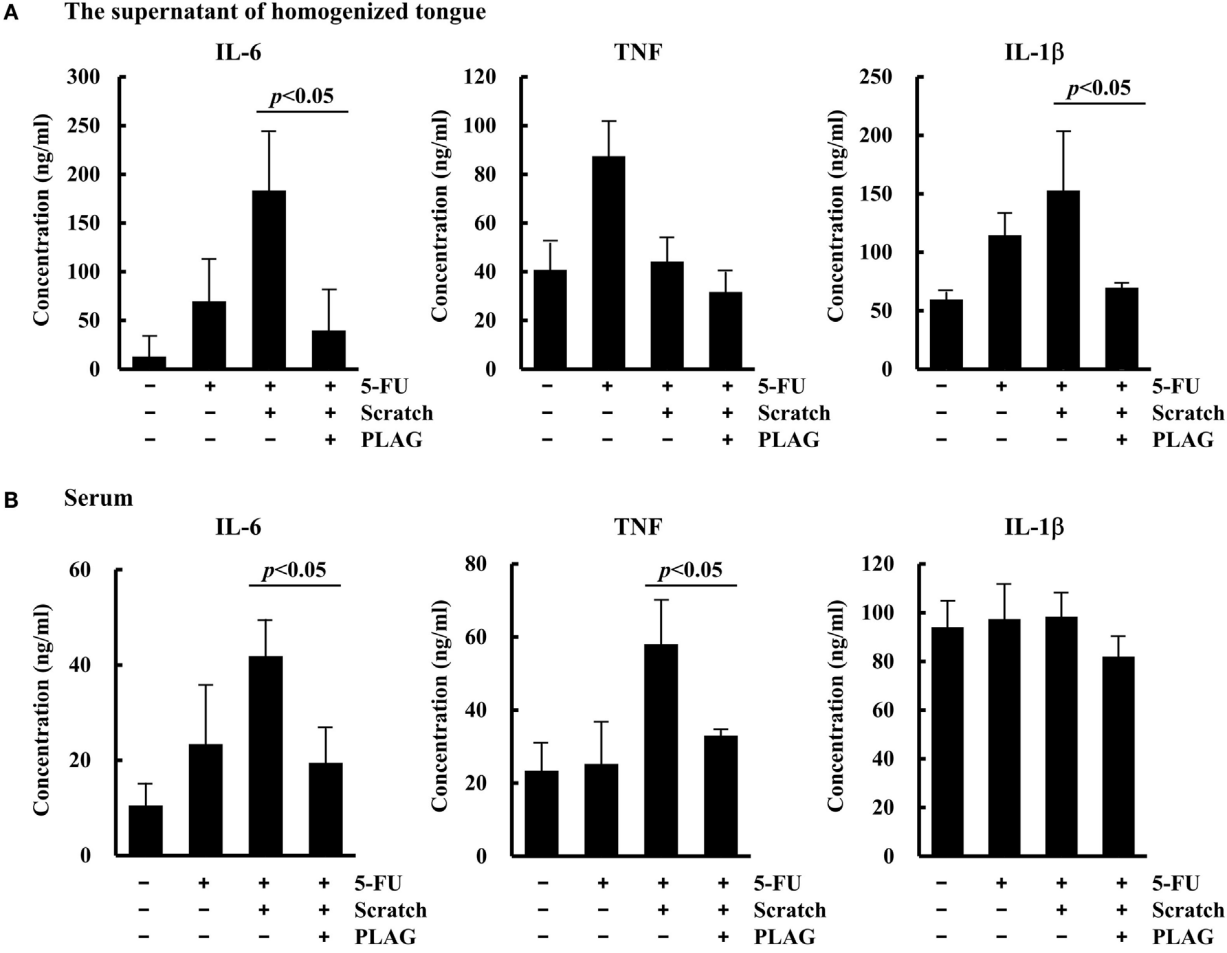
**Levels of proinflammatory cytokines in the tongue and serum**. The groups of mice were treated as described for Figure 5A. On day 15, the tongue and serum were obtained from the mice in each group. The secreted levels of IL-6, TNF, and IL-1β were determined in the supernatant of homogenized tongue tissue **(A)** and serum **(B)** using an ELISA.

To determine whether PLAG has an anti-inflammatory effect in mice with oral mucositis, levels of various chemokines were measured in the supernatant of homogenized tongue tissue and serum. Levels of the representative inflammatory cytokine IL-6 increased as a result of 5-FU/scratching treatment but were significantly reduced in both the tongue (Figure 6A) and serum (Figure 6B) following PLAG administration. PLAG administration also resulted in decreases in the levels of TNF in the serum and IL-1β in the tongue. These data indicate that PLAG plays an anti-inflammatory role and could therefore be a useful therapeutic agent for treating mucositis-associated inflammation.

## Discussion

We previously reported that PLAG administration efficiently blocks neutrophil extravasation and increases the number of circulating neutrophils when used with pegfilgrastim during gemcitabine treatment (14). These data suggest that PLAG exerts a therapeutic effect with pegfilgrastim on chemotherapy-induced neutropenia by regulating neutrophil transmigration. In cancer patients, inflammation is regarded as a critical factor indicative of tumor malignancy or chronic inflammation-induced sepsis (18, 19). To characterize the effect of PLAG on the severity of inflammation associated with chemotherapy, mucositis indexes were evaluated in the induced oral mucositis hamster and mouse models. As shown in Figure 1, mucositis scores and the volume and weight of cheek pouches in the animals subjected to 5-FU/scratching treatment were increased and significantly decreased, respectively, following PLAG administration. This indicates that neutrophil activation for transmigration is induced by 5-FU/scratching treatment and could result in increased neutrophil infiltration with subsequent formation of ulcers (i.e., mucositis). Moreover, histochemical staining data showed that the dermal necrosis induced by 5-FU/scratching was alleviated by PLAG administration (Figure 3). PLAG, therefore, appears to block neutrophil recruitment and minimize the mucositis burden by preventing excessive neutrophil transmigration into the scratched tissues.

Cachexia is a complication associated with both cancer chemotherapy and mucositis. Tumor-induced inflammatory molecules can affect host metabolism, leading to apoptosis (20). Although the incidence of cachexia is high in cancer patients, there is no specific medicine available for treating the condition. PLAG has the potential to not only prevent the weight loss induced by mucositis but also to promote the recovery of lost body weight (Figures 2 and 5). Both the viability and ease of movement in PLAG-treated mice was much better than that of mice subjected to 5-FU/scratching alone. Further studies are needed to reveal how PLAG affects metabolism and physical activity.

A direct association exists between inflammation and the acceleration of cancer malignancy and neutrophil transmigration following chemotherapy (18, 19). Mice treated with 5-FU exhibited diarrhea associated with the significant increases in colon chemokine levels, including levels of CXCL1, 2, and 3 as well as neutrophil recruitment (21). The biomarker for cachexia also included inflammatory cytokines (22). We found that PLAG reduced lipopolysaccharide-induced myeloperoxidase activity and neutrophilic inflammation-related gene expression in an acute lung injury mouse model (data not shown). In this study, we verified that PLAG plays an anti-inflammatory role in chemotherapy-induced oral mucositis and, subsequently, promotes the healing of ulceration and reduces inflammation (Figures 1 and 5). PLAG administration also significantly reduced the expression of inflammatory cytokines (IL-6, TNF, and IL-1β) induced by 5-FU/scratching in the tongue and serum in the mouse model (Figure 6). These data demonstrate that PLAG exerts an anti-inflammatory effect in the induced oral mucositis hamster and mouse models. Therefore, PLAG could be a useful adjuvant for treating chemotherapy-induced side effects *via* modulation of neutrophil migration.

## Author Contributions

JWK and SY designed the project and prepared the manuscript. H-RL and NY performed in vivo assay, ELISA assay, and data collection. The manuscript was completed by H-RL. IHC were performed and analyzed by JHK. Experiments for functional analysis were analyzed by K-YS, H-JK, and M-HK. MYH analyzed the result of animal experiment and provided scientific expertise. All authors discussed the results and commented on the manuscript.

## Conflict of Interest Statement

The authors declare that the research was conducted in the absence of any commercial or financial relationships that could be construed as a potential conflict of interest.
